# Chewing and Swallowing Patterns for Different Food Textures in Healthy Subjects

**DOI:** 10.1155/2023/6709350

**Published:** 2023-06-17

**Authors:** Kartika Indah Sari, Anggun Rafisa

**Affiliations:** Department of Oral Biology, Faculty of Dentistry, Universitas Padjadjaran, Bandung, Indonesia

## Abstract

**Aims:**

This study aimed to determine the patterns of chewing and swallowing in healthy subjects with different food textures.

**Methods:**

This cross-sectional study included 75 subjects who were asked to video record themselves while chewing different food samples of varying textures, including sweet and salty food. The food samples were coco jelly, gummy jelly, biscuit, potato crisp, and roasted nuts. A texture profile analysis test was used to measure the hardness, gumminess, and chewiness of the food samples. Chewing patterns were investigated by measuring the chewing cycle prior to the first swallow (CS1), the chewing cycle until the last swallow (CS2), and the accumulation of chewing time from the first chewing to the last swallowing (STi). Swallowing patterns were evaluated by calculating the swallowing threshold, which is the chewing time/duration prior to the first swallow (STh). The number of swallows for each food sample was also recorded.

**Results:**

There was a statistically significant difference in the CS2 of potato crisps, as well as the STi of coco jelly, gummy jelly, and biscuits between male and female subjects. A significant positive correlation was found between hardness and STh. There was a significant negative correlation between gumminess and all chewing and swallowing parameters, as well as chewiness and CS1. This study also found s significant positive correlation between dental pain, CS1, CS2, and STh of gummy jelly, as well as dental pain and CS1 of biscuits.

**Conclusions:**

Females require longer chewing time for harder foods. Food hardness is positively related to the chewing duration prior to the first swallow (swallowing threshold/STh). Food chewiness has a negative correlation with the chewing cycle prior to the first swallow (CS1). Food gumminess is inversely related to all the chewing and swallowing parameters. Dental pain is associated with an increased chewing cycle and swallowing time of hard foods.

## 1. Introduction

Chewing and swallowing, which occur during eating and drinking, are examples of oral activities that occur throughout life. Chewing, or mastication, is the ability of an individual to process food through a series of masticatory cycles required to comminute and soften the food, transforming it into a bolus suitable for swallowing. Mastication's process of crushing and lubricating food provides information as well as a clear distinction between physiological and pathophysiological conditions [1, 2]. Chewing problems are associated with poor oral health conditions such as tooth loss, ill-fitting dentures, and xerostomia (dry mouth) [3]. Furthermore, because it affects the feeding process, impaired chewing function has been linked to gastrointestinal disorders and malnutrition [4]. Several studies found that more rapid and fewer chewing cycles contributed to an increase in high-energy food consumption and a decrease in vegetable and fruit consumption, which resulted in higher body weight and obesity [5].

Jaw movement, which plays a role in the process of food manipulation during the chewing process, is affected by different food textures [6]. However, previous studies that explored the correlation between food hardness, chewing cycles, and chewing times show some disputes. Several studies found that harder food needs more chewing cycles and more time to be swallowed [7, 8]. Other studies did not find a difference in chewing cycles for different food hardnesses [9–11]. Whereas a study found that harder food needs fewer chewing cycles [12]. These contradictory findings could be explained by the possibility that food has other properties that affect the masticatory response, such as elasticity, gumminess, chewiness, and brittleness [13].

In terms of muscle activities for closing and opening the jaw, the process of bolus formation is adjusted along with the changing textures of food as it is broken down and softened by saliva during the mastication process [14]. During the initial mastication sequence, it was reported that the chewing cycle was higher for harder foods [8, 15]. This obviously affects the overall duration of bolus formation. The chewing rate of foods of varying hardness will become less distinct at the end of the mastication sequence [8]. This demonstrates that when investigating chewing cycles and times, it is critical to note that there is a difference in force and time required at the beginning versus the end of the mastication process. It is very possible that other food textures, in addition to hardness, may influence these variations.

This study aimed to determine the chewing and swallowing patterns of healthy subjects using five sample foods with different textures, such as hardness, gumminess, and chewiness. This study also explored how each food texture affected chewing and swallowing parameters at the beginning of the chewing sequence (prior to the first swallow), as well as the overall cycle and time required for the food to be completely swallowed. This study was conducted outside the laboratory using the video recording method.

## 2. Material and Methods

### 2.1. Study Design and Participants

This cross-sectional study was conducted in November 2021, during the COVID-19 pandemic. This study included 75 healthy subjects who were 2nd-year preclinical dental students (18–20 years old). These students participated in this study as part of an online practicum activity during the implementation of the school-from-home policy.

Subjects were asked to complete a questionnaire that included demographic information (age and sex), weight, height, the Eichner Index's categories of tooth loss, chewing habits (right only, left only, or double-sided), Angle's classifications of malocclusion, and their current experience with dental pain using a numerical rating scale (0–10). Subjects chose the number that best describes the intensity of their pain, with 0 representing “no pain” and 10 representing “the worst pain imaginable.” Because the subjects were dental students who had studied and been trained in determining the classification of malocclusion and tooth loss, they were considered capable of completing the questionnaire themselves.

The inclusion criteria were subjects with Eichner index A and Angle class 1 malocclusion who agreed to participate in the study by signing an informed consent form. Subjects with systemic diseases, orthodontic appliances, or a dental pain scale higher than 2 were excluded.

The subjects were asked to prepare five samples of food selected with varying textures and flavors, including sweet food such as one mini cup of jelly (15 g), one piece of gummy jelly (4 g), and one piece of biscuit (4 g); and salty food such as one slice of original-flavor potato crisp (diameter ±4 cm) and eight medium-sized grains of roasted nuts (±4 g). The brands of the food samples were predetermined, and subjects were instructed to strictly adhere to the brand and quantity of food samples requested. These five food samples have been chosen because they were freely sold and easily accessible during the COVID-19 pandemic.

### 2.2. Food Samples' Texture Profile Analysis (TPA)

**Figure 1:**
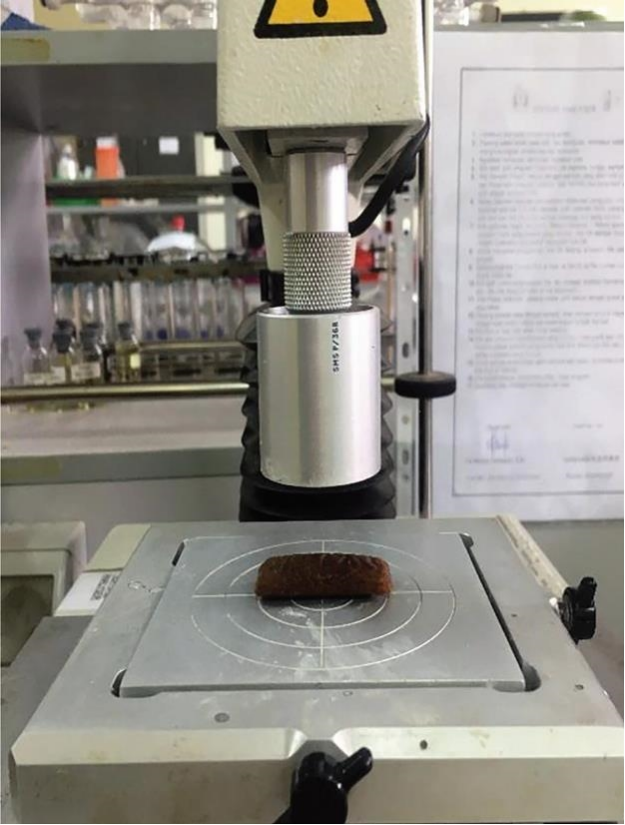
Texture profile analyzer at the Agricultural Technology Laboratory of the Faculty of Agriculture, Universitas Padjadjaran.

The textures of the five food samples were tested with a texture profile analyzer (Stable Micro System Ltd., Surrey, UK); see Figure 1. The tests were performed at the Agricultural Technology Laboratory of the Faculty of Agriculture, Universitas Padjadjaran. TPA is a two-bite test in which the food sample is compressed twice to mimic the action of chewing food. TPA is capable of quantifying multiple textural parameters in a single experiment. However, the textural parameters discussed in this study are hardness, gumminess, and chewiness because the three parameters have the same measuring unit, which is gForce.

Hardness is defined as the force required for a predetermined deformation, and the hardness value is the maximum force that occurs during the first compression on a texture profile analyzer's probe. The energy required to disintegrate a semisolid food before swallowing is referred to as gumminess, whereas the energy required to chew a solid food before swallowing is referred to as chewiness.

### 2.3. Instructions

Subjects were asked to record a video of themselves chewing each food sample. Subjects performed the procedure by sitting upright, leaning back in the chair, and relaxing. The camera was focused on the subjects' faces, with a 20 cm distance between the camera and the face. The heads and eyes of the subjects were pointing forward and parallel to the floor (the horizontal line). Subjects were asked to eat each food sample one at a time (a bite size) in their natural manner. Each food sample was eaten twice, and subjects were asked to rinse their mouths in between.

### 2.4. Variables

Chewing patterns were investigated by measuring the chewing cycle prior to the first swallow (CS1), the chewing cycle until the last swallow (CS2), and the accumulation of chewing time from the first chewing to the last swallowing (STi). Swallowing patterns were evaluated by calculating the swallowing threshold, which is the chewing time/duration prior to the first swallow (STh). One chewing cycle is defined as one movement of opening and closing the jaw into occlusal contact. For each food sample, the number of swallows was also recorded.

Two observers counted the chewing and swallowing patterns by playing back the recorded video at a slower speed and repeating the process twice. To reduce observer bias, the measurement procedures were standardized. The observers had previously been trained to count the chewing cycles, chewing times, swallowing times, and the number of swallows, with greater than 80% agreement in the interrater reliability examination.

### 2.5. Statistical Analysis

All data were analyzed using Microsoft Excel 2016 (Microsoft Corp., Version 16.0, Redmond, WA) and IBM Statistical Product and Service Solutions version 25 (IBM, Armonk, NY). Categorical data are displayed in counts and percentages. The normally distributed data are represented by the mean ± SD, while the nonnormally distributed data are represented by the median (min–max). The Mann–Whitney test was used to compare chewing and swallowing patterns based on sexes for nonparametric data and the independent *t*-test for parametric data. Spearman's rank correlation coefficient was used to determine the relationship between variables in the study, such as the TPA results and dental pain with chewing and swallowing parameters. The statistical analysis was performed at the significance level of *p* < 0.05.

## 3. Results

Table 1 shows the basic characteristics of the subjects in the study. The majority of subjects were female, 19 years old, chewed on both sides, had a normal BMI category, and had no current dental pain (scale = 0).

The textures of food samples, as determined by the TPA test, are shown in Table 2. The food sample with the highest hardness value was biscuit, and the food sample with the highest gumminess and chewiness value was gummy jelly. Potato crisp had the lowest hardness and chewiness values, while roasted nuts had the lowest gumminess value.

Table 3 displays the CS1, CS2, STh, STi, and the number of swallows values of five food samples across all subjects as well as in male and female subjects. There was a statistically significant difference in the CS2 of potato crisps, as well as the STi of coco jelly, gummy jelly, and biscuits between male and female subjects.

**Figure 2:**
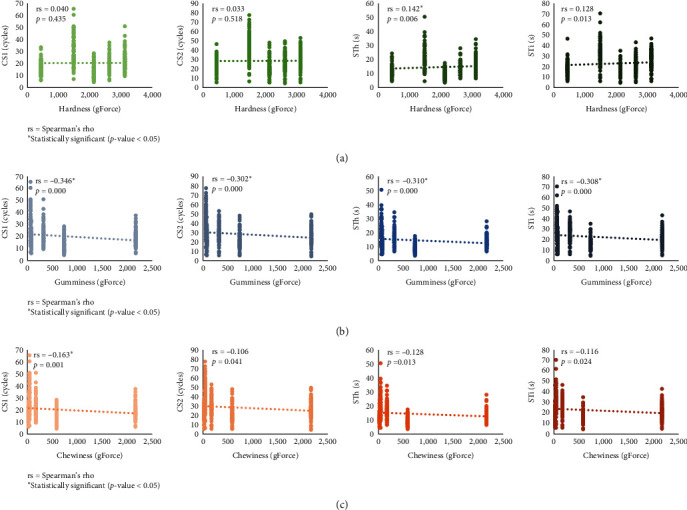
The comparison of chewing and swallowing parameters of food samples according to their hardness (a), gumminess (b), and chewiness (c) values. The vertical left axis displays the values of chewing cycles (CS1 and CS2), swallowing times (STh), or chewing times (STi). Each parameter's linear trendline is represented by a dotted line. The correlation between each parameter and the food sample's hardness, gumminess, or chewiness was measured by Spearman's rank correlation coefficient.

Figure 2 compares the chewing and swallowing parameters of five food samples based on their TPA test results, which are hardness, gumminess, and chewiness.  Figure 2(a) shows a trendline of chewing and swallowing parameters based on hardness with little slope and a tendency to be horizontal, indicating a constant relationship. The statistical analysis discovered a positive correlation, but it was only significant in the relationship between hardness and STh. Chewing and swallowing parameters based on food gumminess (Figure 2(b)) and chewiness (Figure 2(c)) show a trendline with a negative slope. A significant negative correlation was found between gumminess and all chewing and swallowing parameters, as well as chewiness and CS1.

The correlation between dental pain and chewing and swallowing parameters of food samples is shown in Table 4. There is a significant positive correlation between dental pain, CS1, CS2, and STh of gummy jelly. This study also found a significant positive correlation between dental pain and CS1 of biscuits.

## 4. Discussion

### 4.1. Chewing and Swallowing Patterns Based on Sex

Females required more total chewing cycles (CS2) of potato crisp than males, although there was no difference in the chewing cycle prior to the first swallow (CS1). It indicates that males and females had a similar number of chewing cycles of potato crisp at the beginning of the masticatory sequence, but females required more cycles until the food was completely swallowed. TPA results show that potato crisp has the lowest hardness and chewiness values, as well as the second lowest gumminess value. As a result, this difference could be attributed to additional food textures that were not assessed in this study, such as springiness, adhesiveness, cohesiveness, and resilience.

Another explanation for the variation could be the flavor of the food. Compared to other food samples in this study, potato crisp had a higher salt content. Previous studies have shown that salty foods stimulate the production of saliva, which can shorten the chewing cycle and duration [16, 17]. Furthermore, in this study, the BMI may affect the total chewing cycles. The majority of male subjects (46.15%) were classified as overweight, whereas the majority of female subjects (66.13%) were classified as normal weight. Several prior research have shown that subjects with a higher BMI have fewer chewing cycles [18, 19]. On top of that, more research is required to test these possibilities.

Female subjects also required a longer total duration to chew (STi) coco jelly, gummy jelly, and biscuits. These foods have the highest values of hardness among the five food samples, which may explain why female subjects required longer total chewing time [7, 8]. The tendency of males to have shorter chewing times than females has been reported before [20]. Males were found to have stronger masticatory force than females and to be fast eaters regardless of whether they were normal weight, overweight, or obese. [21].

### 4.2. The Effect of Different Food Textures on Chewing and Swallowing Patterns

This study found that harder food needs longer time to be manipulated at the beginning of the masticatory sequence (prior to the first swallow/STh). However, food hardness has no effect on the total chewing time required to completely swallow the food (STi). These results confirm the findings of the previous studies [8, 15]. The manipulation of hard food involved a slower process at the beginning of the chewing sequence to overcome food resistance. After the first swallow, food manipulation will become easier since the food has been sufficiently broken down and lubricated by saliva, allowing even hard food to be ingested more quickly [8].

Only the chewing cycle prior to the first swallow (CS1) was found to be inversely linked with food chewiness. It implies that chewy foods require fewer chewing cycles at the beginning of the masticatory sequence but a relatively constant number of chewing cycles throughout. It also indicates that the more energy necessary to chew food before the first swallow, the fewer chewing cycles will be involved. These findings opposed prior research on the subject, which discovered that foods with lower chewiness values had fewer chewing cycles [22, 23]. This may be due to the prior study's utilization of different food samples with a wide range of chewiness values.

According to the current study, food gumminess was the characteristic that determines all chewing and swallowing patterns. The chewing cycle and duration, as well as the swallowing threshold, were found to be inversely related to the gumminess value. The gummier the food, the fewer cycles and time necessary to chew it, whether at the beginning or the end of the masticatory sequence.

This study suggests that hard foods, such as biscuits, are more suitable for assessing masticatory force. Masticatory force is the value of peak in the initial bite at maximum compression in TPA and is directly proportional to swallowing time (swallowing threshold). Due to the association discovered with all observed chewing and swallowing patterns, gummy jelly, as a food with a high gumminess and chewiness value, is more suitable to be used to objectively evaluate masticatory performance. The unique properties of gummy jelly have been exploited in previous studies involving the objective measurement of masticatory performance [24–26]. It was also discovered that as gelatin content increased, the integral value of masseter muscle activity increased considerably [25].

### 4.3. The Effect of Dental Pain on Chewing and Swallowing Patterns

The current study also found a statistically significant correlation between dental pain, chewing cycle, and swallowing time of gummy jelly, as well as dental pain and chewing cycle prior to the first swallow of biscuit. Compared to other food samples in this study, gummy jelly had the highest gumminess and chewiness values, and biscuit had the highest hardness value. This once again provides evidence that the texture and characteristics of food can greatly influence the process of chewing and swallowing. Because tooth contact is required during mastication, the presence of dental pain undoubtedly causes difficulty in performing the normal mastication process [27]. Modification of food properties that promotes the growth and development of the masticatory system may potentially be an alternative for anticipating dental occlusion disturbance [28].

## 5. Conclusion

Females require longer chewing time for harder foods. Food hardness is positively related to the chewing duration prior to the first swallow (swallowing threshold/STh). Food chewiness has a negative correlation with the chewing cycle prior to the first swallow (CS1). Food gumminess is inversely related to all the chewing and swallowing parameters. Therefore, gummy jelly, as a food with a high gumminess and chewiness value, is more suitable to be used to objectively evaluate masticatory performance. Dental pain is associated with an increased chewing cycle and swallowing time of hard foods.

## Figures and Tables

**Table 1:** The basic characteristics of subjects.

No.	Variable	Total subjects (*n* = 75)	Frequency (%)
1.	Age (years)		
	18	11	14.7
	19	48	64.0
	20	14	18.7
	21	2	2.7

2.	Gender		
	Male	13	17.3
	Female	62	82.7

3.	Chewing habits		
	Right only	6	8.0
	Left only	4	5.3
	Double-sided	65	86.7

4.	Body mass index (BMI)		
	Underweight	17	22.7
	Normal	45	60.0
	Overweight	11	14.7
	Obese	2	2.7

5.	Dental pain scale		
	Scale 0	51	68.0
	Scale 1	17	22.7
	Scale 2	7	9.3

*n* = count.

**Table 2:** TPA results of food samples.

No.	Analysis parameters	Food samples					Units
		Potato crisp	Coco jelly	Gummy jelly	Roasted nut	Biscuit	
1.	Hardness	432.7	2,128.8	2,626.4	1,482.0	3,123.0	gForce
2.	Gumminess	75.1	733.4	2,170.7	60.2	319.8	gForce
3.	Chewiness	22.6	585.2	2,165.5	42.6	169.8	gForce

**Table 3:** The chewing and swallowing parameters of food samples based on sex.

No.	Food samples	CS1 (cycle)				CS2 (cycle)				STh (s)				STi (s)				Number of swallows			
		Total subjects (*n* = 75)	Male (*n* = 13)	Female (*n* = 62)	*p*	Total subjects (*n* = 75)	Male (*n* = 13)	Female (*n* = 62)	*p*	Total subjects (*n* = 75)	Male (*n* = 13)	Female (*n* = 62)	*p*	Total subjects (*n* = 75)	Male (*n* = 13)	Female (*n* = 62)	*p*	Total subjects (*n* = 75)	Male (*n* = 13)	Female (*n* = 62)	*p*
1	Potato crisps	16.8 ± 5.7	14.8 ± 4.4	17.3 ± 5.9	0.145	21.5 (5.5–46.5)	18.6 ± 5.4	23.9 ± 8.3	0.025 ^*∗*^	11.0 (4.5–24.3)	9.8 (5.0–22.0)	11.8 (4.5–24.3)	0.118	18.3 ± 6.5	15.3 ± 5.0	19.0 ± 6.7	0.051	2.0 (1.0–9.5)	2.0 ± 0.62	2.4 ± 1.3	0.228

2	Coco jelly	13.8 ± 5.9	13.0 (5.5–26.5)	13.0 (4.5–28.5)	0.702	20.5 (5.5–48.0)	18.1 ± 6.1	22.9 ± 8.9	0.057	9.0 (3.5–17.5)	8.9 ± 3.0	9.4 ± 3.1	0.57	16.3 ± 5.4	13.0 ± 3.6	17.0 ± 5.5	0.013 ^*∗*^	2.0 (1.0–6.0)	2.3 ± 1.3	2.5 ± 0.7	0.094

3	Gummy jelly	18.8 ± 6.7	17.4 ± 5.6	19.2 ± 6.9	0.375	26.8 ± 9.16	22.8 ± 7.0	27.6 ± 9.4	0.076	12.5 (0.0–28.0)	11.8 ± 3.3	14.0 ± 4.7	0.092	21.4 ± 7.0	16.8 ± 4.6	22.4 ± 7.0	0.006 ^*∗*^	2.0 (1.0–5.0)	2.1 ± 0.7	2.5 ± 01.0	0.139

4	Roasted nuts	29.8 (7.0–65.5)	26.8 (16.5–65.5)	30.2 (7.0–60.5)	0.701	42.6 ± 14.7	41.6 ± 14.1	42.8 ± 15.0	0.793	19.3 (4.5–50.5)	18.3 (11.0–37.0)	20.0 (4.5–50.5)	0.419	32.6 ± 11.3	29.9 ± 9.6	33.2 ± 11.7	0.325	3.0 (1.0–14.5)	2.8 ± 1.8	3.3 ± 2.1	0.545

5	Biscuit	21.5 (9.5–51.0)	20.4 ± 8.3	22.4 ± 7.5	0.385	29.2 ± 9.6	26.0 ± 8.4	29.9 ± 9.8	0.174	16.9 ± 5.8	14.8 ± 6.9	17.3 ± 5.5	0.143	26.4 ± 8.1	21.7 ± 6.1	27.4 ± 8.1	0.02 ^*∗*^	3.0 (1.0–9.0)	2.0 (1.0–4.0)	3.0 ± 1.3	0.193

*n* = count. The normally distributed data are represented by the mean ± SD, while the nonnormally distributed data are represented by the median (min–max).  ^*∗*^Statistically significant (*p*-value < 0.05) according to the Mann–Whitney test for nonparametric data and the independent *t*-test for parametric data.

**Table 4:** Correlation between dental pain with chewing and swallowing parameters of food samples.

No.	Food samples	The correlation between dental pain with									
		CS1		CS2		STh		STi		Number of swallows	
		rs	*p*	rs	*p*	rs	*p*	rs	*p*	rs	*p*
1	Potato crisps	0.172	0.137	0.157	0.177	0.160	0.168	0.124	0.290	−0.053	0.659
2	Coco jelly	0.140	0.231	0.098	0.400	0.118	0.310	0.128	0.271	0.096	0.418
3	Gummy jelly	0.329^*∗*^	0.004	0.233^*∗*^	0.043	0.313^*∗*^	0.006	0.177	0.126	0.042	0.722
4	Roasted nuts	0.147	0.205	0.111	0.339	0.121	0.298	0.071	0.540	−0.194	0.100
5	Biscuit	0.258^*∗*^	0.024	0.196	0.090	0.195	0.097	0.174	0.135	−0.065	0.586

rs = Spearman's rho.  ^*∗*^Statistically significant (*p*-value < 0.05).

## Data Availability

The datasets used and/or analyzed during the current study are available upon reasonable request.

## Additional Points

*Study Limitations*. This study utilized commercial foods that were chosen because they were widely available during the COVID-19 outbreak. However, these foods may have other features that cannot be controlled, such as food preparation, oil content, and chemicals or nutrients that may impact chewing and swallowing processes. Furthermore, this study focused solely on three different types of food textures. In future research, variables, such as springiness, adhesiveness, cohesiveness, robustness, and water content, may be explored to further expand our understanding.
